# COSMOS: Computational Shaping and Modeling of Musical Structures

**DOI:** 10.3389/fpsyg.2022.527539

**Published:** 2022-05-27

**Authors:** Elaine Chew

**Affiliations:** ^1^STMS, CNRS, IRCAM, Sorbonne Université, Ministère de la Culture, Paris, France; ^2^Department of Engineering, King's College London, London, United Kingdom

**Keywords:** music performance, computational modeling, technology, citizen science, musical structure, cardiac arrhythmia

## Abstract

This position paper makes the case for an innovative, multi-disciplinary methodological approach to advance knowledge on the nature and work of music performance, driven by a novel experiential perspective, that also benefits analysis of electrocardiographic sequences. Music performance is considered by many to be one of the most breathtaking feats of human intelligence. It is well accepted that music performance is a creative act, but the nature of its work remains elusive. Taking the view of performance as an act of creative problem solving, ideas in citizen science and data science, optimization, and computational thinking provide means through which to deconstruct the process of music performance in scalable ways. The method tackles music expression's lack of notation-based data by leveraging listeners' perception and experience of the structures elicited by the performer, with implications for data collection and processing. The tools offer ways to parse a musical sequence into coherent structures, to design a performance, and to explore the space of possible interpretations of the musical sequence. These ideas and tools can be applied to other music-like sequences such as electrocardiographic recordings of arrhythmias (abnormal heart rhythms). Leveraging musical thinking and computational approaches to performance analysis, variations in expressions of cardiac arrhythmias can be more finely characterized, with implications for tailoring therapies and stratifying heart rhythm disorders.

## 1. Introduction

At first blush, music performance, musical structures, and cardiac arrhythmia may seem like words that should not be uttered in the same sentence, much less discussed in the same paper. However, the shared musical structures between music and cardiac signals, and the underlying constraints of human physiology mean that analytical approaches for characterizing music performance can also be applied to cardiac recordings. Here, I shall present an overview of these topics as they relate one to another, including the motivation for focusing on musical structures, particularly structures created by performers in music communication; citizen science and computational approaches to finding and exploring such structures; why and how these techniques apply to cardiac information; and, the practical implications of these theoretical connections. The discussions introduce and are circumscribed by the scope of COSMOS, a 5 year European Research Council (ERC) project on Computational Shaping and Modeling of Musical Structures.

Performance and the expressivity that it entails underlies almost all the music that we hear, but the study of performance through technological means is a relatively recent phenomenon (Juslin et al., 2001; Widmer et al., 2003; Widmer and Goebl, 2010; Devaney et al., 2011; Kirke and Miranda, 2013; Cancino-Chacón et al., 2018). This is due in part to the fact that the problems of performance are not well understood nor well defined. To bridge this gap, I present here a view of music performance as a creative problem-solving task centered on the search for and communication of musical coherence or structure. From this vantage point, choices in performance are driven by the human desire for coherence, which necessitates the making and shaping of structure in music. Thus, performance is tightly linked to musical structure, broadly defined, and successful performances are marked by clear communication of structure and intent.

Music structure most frequently refers to sectional, repeating forms such as sonata form or ABA structure (Stein, 1979; Paulus et al., 2010; Wu and Bello, 2010; Davie, 2014). Here, the term structure is used more generally to refer to all forms of organization of musical matter, from surface features to deeper ones, including musical entities and boundaries, and movements and arrivals. For example, long-term intensity modulation constitutes a form of structure, as do articulations to mark local note groupings, note weightings indicating an upbeat or downbeat, or subtle changes of timbre amidst a sustained note. These structural features serve the function of generating coherence in music. With this definition of musical structure in mind, the work of performance then becomes one of finding, even creating, musical coherence.

The ways in which performance ties in to this broad notion of musical structure provides avenues for computational scientists and music technologists to more closely engage with research on performed music and music performance. This suggests novel approaches to thinking and asking questions about musical structure, generating new ways to problematize the concept of musical structure, and to find this structure. Because the task of performance can be framed as the finding of musical coherence, solving for musical structures (broadly defined) thus accomplishes the essential work of performance and interpretation.

A key idea here is one of re-thinking and re-framing problems of music performance so that they can be formulated in smart and authentic ways that interface smoothly with technologies. Finding the right problem is frequently overlooked as being at least as important as solving the problem itself (Pounds, 1966). After observing managers spending substantial amounts of time solving problems defined by themselves or by others, Pounds proposed a scientific alternative to this frenetic approach: “until one has a fairly reliable model of the environment, it is not only foolish but perhaps even dangerous to take action when its effect cannot be predicted” (p.33). Furthermore, “problem definition cannot precede model construction” (p.35). Thus, one needs to first understand the nature of performance before tackling technological innovations that interface with music performance. This advice is especially relevant today as computers are now capable of solving problems of ever increasing complexity.

The research directions proposed in this position paper draw from a range of disciplines: music engagement and musical structure in music information research, creative thinking and problem solving in cognitive psychology, the citizen science movement for scientific research, computer games for shaping performance, duality and inverse problems in applied mathematics, and computational thinking in computer science. Inspired by the growing citizen science movement (Vohland et al., 2021), an approach is proposed here to involve citizens—experienced musicians, retired performers, everyday music listeners—in the scientific endeavor of de-constructing the problems of music performance. To evaluate the amount of information provided by citizen scientists and to model and solve problems at scale, computational thinking (Wing, 2006) is invoked to ensure accuracy, efficiency, and scalability. To focus on modeling the decisions and perceptions of individuals, an inverse problem approach (Vogel, 1987) is proposed through a duality paradigm. To enhance learning by doing (Anzai and Simon, 1979) and drawing upon a wealth of music games for shaping performance, everyday citizens will be encouraged to engage in the task of exploring the space of possible interpretations by designing and re-shaping performances and by thinking about their decisions.

The methods stand to increase the understanding of how performers interpret musical sequences and the reasons for their choices, and to open up new avenues for exploring potential or as-yet-unknown interpretations of musical sequences. This can lead to knowledge of the strategies employed in expressive performance, thereby allowing for the devising of unconventional new performances that are nevertheless musically convincing.

Another goal of this position paper is to highlight the translational potential of techniques developed for analysing and characterizing music performance in music-like sequences such as electrocardiographic traces of cardiac arrhythmias. Arrhythmia is the clinical presentation of abnormalities in cardiac electric pulse generation or impulse conduction through heart muscle (Dowd, 2007; Karpawich, 2015). These anomalies can result in rhythms that are slow or fast, regular or irregular, and that originate from different anatomical sites in the heart. The paper will discuss the commonalities between music and arrhythmic heart signals which make cardiac information amenable to music structure analysis. Current medical categorizations of cardiac arrhythmias are limited and based primarily on rate, source, and regularity, often providing little information as to the symptoms and outcomes. Over the centuries, musicians have developed a rich vocabulary to describe rhythm-based variations. This knowledge can be transferred to characterize cardiac rhythm variations, to provide better descriptors to personalize diagnostics and therapeutic decisions. These theoretical connections extend the impact of methodologies developed for understanding music performance to computational cardiology. They also broaden the scope of computational performance analysis to studies on the interactions between music and the heart.

This position paper describes the aims and scope of COSMOS—Computational Shaping and Modeling of Musical Structures—a 5-year research project funded by the European Research Council (ERC). The project aims to study musical structures as they are created in performance and in cardiac arrhythmias using data science, citizen science, and optimization. Like in Widmer (2017)'s manifesto, the problems identified here and their scope are far greater than can be tackled by one project alone. Thus, this is also a call to the community to join in the efforts to tackle these research issues.

The remainder of the paper is organized as follows: Section 2 lays the groundwork for the research directions of COSMOS; Section 3 provides evidence for the transfer of music structure analysis techniques to ECG sequences; Section 4 outlines the COSMOS objectives and research themes, with conclusions in Section 5.

## 2. Issues of Performance and Structure

This section presents the tenets of the proposed research directions, and reviews supporting evidence for them. An essential guiding principle of performance is the creating of engaging experiences; Section 2.1 reviews the growing body of research on music engagement and the importance of musical structure in this context. In Section 2.2, performance is described as a form of creative thinking, and as such can be regarded as an act of problem solving. A description of the use of citizen science, a.k.a. volunteer thinking, in other domains and in music follows in Section 2.3. Section 2.4 reviews the use of metaphors in games for experimenting with performance decisions. The concept of duality is introduced in Section 2.5, a re-framing of problems in computational music structure analysis. Finally, Section 2.6 argues for computational thinking that prioritizes accuracy, efficiency, and scalability in models.

### 2.1. Music Engagement: Interrogating the Musical Experience

Global digital media revenues in excess of one trillion USD have bolstered the demand for music information technologies. According to the International Federation of the Phonographic Industry (IFPI, 2020)'s most recent Global Music Report, worldwide digital revenues for recorded music were up by 8.2% in 2019. The people polled spent 18 h per week listening to music, which equates to 52 3-min songs daily. Streaming revenues rose by 22.9%, with Spotify alone having 345 million users and 115 million paying subscribers by the end of 2020 (Music:)Ally, 2021). Even relative newcomer Apple Music has 72 million paying subscribers in June 2020 (Statistica, 2021), and had acquired the music recognition app, Shazam, which had surpassed one billion downloads (Smith, 2020). With such widespread access to large digital music collections, there is substantial interest in issues of music engagement and the musical experience. The important question thus arises:

*How do people engage with the music that they hear? What music features do they attend to and what music structures do they perceive?*

Large-scale digital music datasets have spurred interests in **how people engage with music collections**, leading to research in hit song identification (Pachet, 2002; Pachet and Roy, 2008), where top Billboard positions have been correlated with audio features (Herremans et al., 2014), instrumentation (Nunes and Ordanini, 2014), and lyric repetition (Nunes et al., 2015). In a first ever large-scale ecological study relating when users choose to send a Shazam query on a popular song in relation to its popularity and song structure, Kaneshiro et al. (2017) reports an increase in queries at the onset of the vocals and the first occurrence of the chorus, this changes as a function of the song's popularity or listener exposure to the song. In the general realm of studies on what kinds of features attract listeners and stand out from other music content, research on musical “hooks” in popular songs have found that they tend to occur at structural segmentation boundaries (Burns, 1987; Mercer-Taylor, 1999; Burgoyne et al., 2013), which are linked to moments of change in harmony, melody, timbre, and rhythm (Smith et al., 2014). Empirical studies of musical hooks (Burgoyne et al., 2013) and the related earworms (Williamson and Müllensiefen, 2012) form first steps toward the automated identification of these experiential features (van Balen et al., 2015).

Large-scale corpus studies of music structure as it relates to music experience have focused primarily on popular music, due in part to the available datasets, the computational challenges in automating the cognition of music structures such as beat in classical art music, and the time and labor required to create meaningful manual annotations. While the same can be said of many world musics, room remains for work aiming **to address the gap in population-scale performed music research in classical music**.

The temporal nuances in classical (Western art concert) music, including its contemporary developments, make it particularly challenging for machine analysis. Automatic techniques that speed annotation like computer-based tempo tracking work poorly on classical datasets (Grosche et al., 2010), where movements in tempo are much more volatile than in popular music, which are frequently temporally constrained by the use of click tracks. The alternative, manual tapping to create beat annotations, does not scale, which is why initially beat annotations were available only for a small subset of the Mazurka dataset of the CHARM Project (www.mazurka.org.uk), which had amassed 2500 recordings of 49 Chopin Mazurkas (Sapp, 2007, 2008). A workaround to this problem is to painstakingly manually annotate one recording of a piece, and transfer the annotations to others of the same piece after aligning all other recordings to the reference recording (Wang et al., 2016), then manually checking the transferred annotations. Using this technique, my lab has produced beat and loudness information for 2,000 recordings in the Mazurka dataset to form the MazurkaBL dataset (Kosta et al., 2018) (github.com/katkost/MazurkaBL), which has been used in research on performed loudness (Kosta et al., 2015, 2016).

Not only is extracting the beats themselves difficult, assigning meaning to expressive nuances is also challenging. Sometimes, phrase arching can be clearly demarcated through increasing and decreasing beat rates, and sometimes the beat rate will be entirely meaningless with respect to phrase, with changes in loudness being the main indicator (Cheng and Chew, 2008). This can be the result of performance conventions, or the performer's idiosyncratic decisions. Sometimes, a note elongation signifies a harmonically important note, and sometimes it marks the start of a motif; the start of a motivic grouping can also be demarcated using articulation rather than time. And, sometimes, extreme elasticity of pulse denotes a major tipping point (turning point) (Chew, 2016) in the piece, and sometimes it is simply the slow down at the end of a phrase or piece. The reasoning can be inferred from data with the help of human annotations of music structure.

The limited number of music datasets with structural annotations focus on compositional structure, rather than the felt or experienced structures, although the two are necessarily related. Leveraging basic skills of noting boundaries between sections and assigning labels that indicate which sections contain similar material, the SALAMI (Structural Analysis of Large Amounts of Musical Information) Project (DDMAL, 2019) (ddmal.music.mcgill.ca/research/salami) employed music students at the McGill Schulich School of Music to create 2,400 structural annotations of almost 1,400 musical recordings of popular, jazz, classical, and world music (Smith et al., 2011); variability in listener annotations was addressed by having two students annotate each recording. Other datasets with structure annotations include Tampere University of Technology's TUTstructure07 dataset (www.cs.tut.fi/sgn/arg/paulus/TUTstructure07_files.html), which contains 557 sectional annotations of mainly popular music, and AIST's 285 beat-melody-chorus annotations of the RWC Music Database (Goto, 2006), a quarter of which is jazz and classical music.

*The focus here is on structures felt and experienced, musical analogs of elemental categories such as thresholds and change points, stability and instability, to inform music engagement research*.

Music listening is almost universal amongst humanity. Sloboda (2008) has argued that it is the experience of music that must be studied in order for us to discover its true impact. With some induction, experts and non-professionals alike can draw from their own experiences to provide meaningful input on such experiential structures. Classical music serves as the starting point for our investigations, but other kinds of music also grapple with similar perceptual and structural issues, and the methodologies worked out as a result of this research will also benefit, for example, studies of music made with sounds rather than notes, music without a clear pulse, and music without a notated score.

### 2.2. The Science of Performance: Performance as Problem Solving

Many consider music performance, with its on-the-fly calculations and decision making, to be one of the most breathtaking feats of human intelligence. That music performance is a creative act is no longer a disputed fact. In recent years, musicological views have shifted from music as writing to music as performance (Cook, 2013).

**Figure 1:**
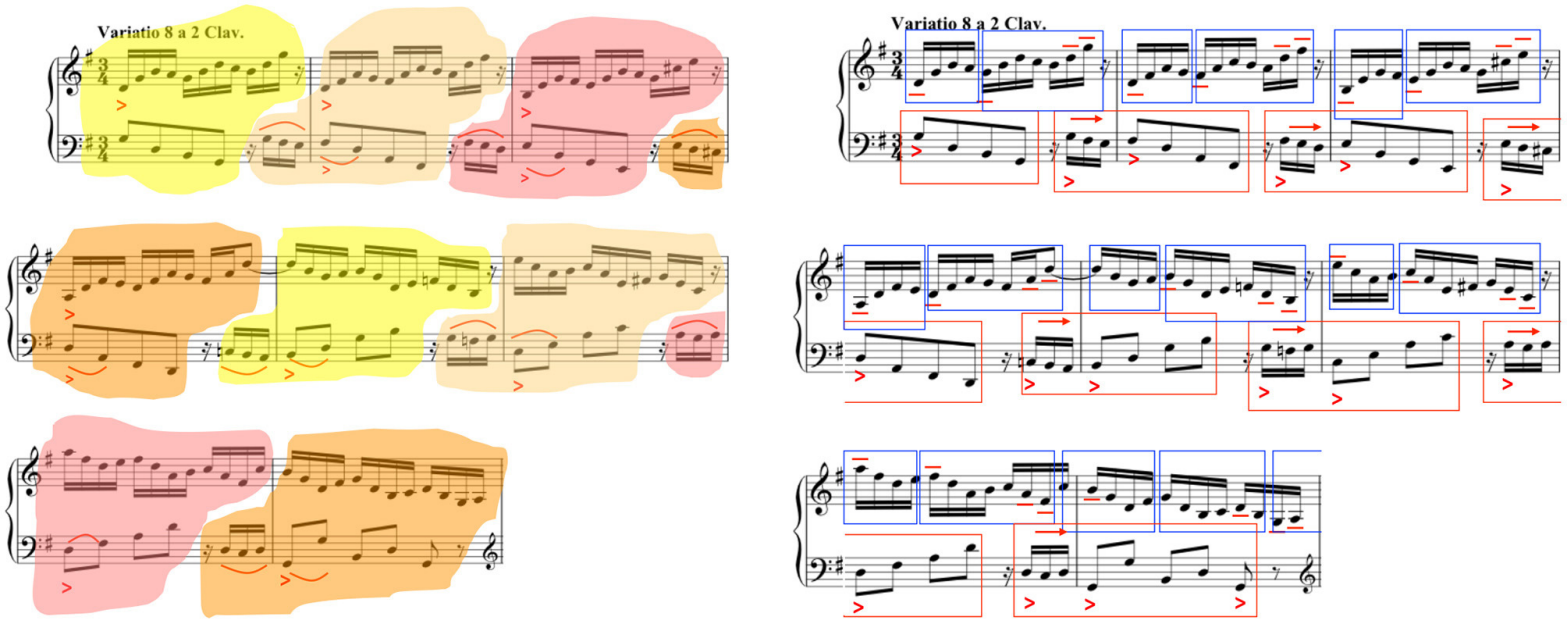
Grouping structures in Glenn Gould's recordings of Goldberg Variation 8 (left) in 1955—see vimeo.com/159472151—and (right) in 1981—see vimeo.com/159488217. Reproduced from Chew (2017).

The written score represents only selected aspects of music knowledge and experience, and often in reduced and approximate form, which means that the information is not only inexact, but is an abstraction of the actual experience (Frigyesi, 1993). Notes are played longer or shorter than indicated (Cook, 2008); pitches are often ornamented in practice (Leech-Wilkinson, 2006; Yang et al., 2013, 2015); and, notated dynamics are frequently not what they seem (Kosta et al., 2015). The same score can yield strikingly different performances—a famous example being Glenn Gould's 1955 and 1981 recordings of Bach's *Goldberg Variations* (see Figure 1)—further evidence of not only the gap between abstract representation and actual performances, but also the creative paths open to the performer. Comparative analyses of multiple performances of the same pieces abound (Cook, 2007; Sapp, 2007, 2008; Rink et al., 2011; Chew, 2012; Kosta et al., 2016), but the nature of the creative work of performance remains elusive. The questions remain:

*What is the nature of the creativity in performance? What is the reasoning behind particular interpretations of a piece of music? What work do performers do to create novel and moving experiences for listeners?*

Any form of **creative thinking can be viewed as a form of problem solving** (Gilhooly, 1996). While little has been written on music performance as problem solving—the nature of the problems to be solved, beyond skill acquisition and sequence planning, see Drake and Palmer (2000), still begs clarification—music composition has long been associated with problem solving (McAdams, 2004) and the creativity it entails. Creative thinking in music composition has been the subject of numerous studies, which Collins (2005) categorizes as product-based, where features of the composition serve as evidence of the cognitive process, or process-based, which focuses on behaviors and thought processes in the act of composing (Webster, 1992). Composition has been likened to mathematical proof or the discovery of mathematical facts (Coxeter, 1968), a particular form of creative problem solving, where the quality of a solution is judged by Hardy (1940)'s principles of *unexpectedness* (novelty), *inevitability*, and *economy*. The same principles apply to the evaluation of composition solutions as well as performance solutions found through creative problem solving. If one views Gould's 1955 and 1981 recordings as two possible solutions to a musical puzzle, what are other ways to devise viable performance solutions that are also unexpected, inevitable, and elegant?

Adopting the view of **music structure as constructed in performance** (Rink, 2016), the making and shaping of music structures thus becomes an important part of the creative work of performance. Here, a structural approach to playing is not antithetical to a rhetorical approach (Cook, 2011) for a rhetorical approach, as in speech, also conveys structure as is broadly defined. That composers and improvisers create music structure may be given. But at first blush the idea that performers and listeners are also important makers of music structure may seem controversial. That **structure is an emergent property of musical thinking and reasoning** is an idea that is at the forefront of musical thought in music education (Bamberger, 2013) and musicology (Rink, 2016), but the idea has not been explored in depth in music research or MIR, nor exploited in computer models of music structures. Recent work (Chew, 2016, 2017) has only begun to theorize how this can be done.

Like in Degas' famous quote, “Art is not what you see, but what you make others see,” (Gammell, 1961) performance offers a window into the performer's understanding of music structure; because it provides a means of hearing structure, it is about the structures that a performer must make others hear. Decisions on how to convey the music structures follows an assessment of the structures in a piece of music (Clarke, 2002; Rink, 2002), for example, deciding whether to delineate the tonal structures of a piece or to highlight the inevitable descent of a bass line or to demarcate the repetition structures will lead to different performance solutions (Chew, 2017). The logic of the choices can be de-constructed *post-hoc* from recorded performances by comparing the structures heard and experienced with structures inherent in the composition, or the process of making performance decisions can be analyzed through the documenting of thought processes as performed structures are being constructed.

Because performance studies is young, in comparison to studies of composition, and the encoding of information on music performance is still in its infancy, the creative thinking in music performance has not been subject to the same degree of scrutiny or analysis. The Beethovens Werkstatt project (beethovens-werkstatt.de) is implementing long-term plans to explore Beethoven's work methods by studying Beethoven's manuscripts complete with re-worked, corrected, and discarded passages. In a reversal of convention, turning performance into writing, in “Practicing Haydn” (Chew, Child, Grønli 2013, vimeo.com/109998951), my sight-reading is transcribed into a performable score, complete with starts and stops, repetitions, and corrections, revealing the cognitive process of constructing a performance. Taking this idea further, free rhythms in performance are transcribed for close examination and cross-comparison in Chew (2018).

### 2.3. Citizen Science: Harnessing Volunteer Thinking for Music Research

In the **citizen science** movement, large numbers of non-professionals with Internet access have been mobilized to assist in authentic, large-scale scientific research. Citizen science has supported projects in fields ranging from astronomy to zoology. Ordinary citizens perform tasks such as classifying galaxies (Raddick et al., 2010), folding proteins (Cooper et al., 2010), tracking migratory behavior (Dickinson et al., 2012), and identifying animal calls (Shamir et al., 2014). These efforts have led to significant scientific discoveries and peer-reviewed publications (see zooniverse.org/about/publications).

Citizen science projects serve not only as platforms for public engagement, they also provide opportunities for learning and creativity (Jennett et al., 2016). In Galaxy Zoo, volunteers (zooites) have discovered new galactic objects like the voorwerpen and Green Peas, impacting astronomers' understanding of the evolution of galaxies (Clery, 2011). Close to the ideas of understanding and designing structures, Foldit (fold.it) participants learn through tutorials the principles of protein folding then compete, as individuals or in teams, to find the best protein folding structures for unsolved sequences. Protein folding belongs to the most difficult class of computational problems that are described as NP-complete, meaning a solution to a large-enough problem can take more than several lifetimes to compute. Collaboratively, Foldit players discovered the protein folding structure of the HIV enzyme within only 3 weeks when the problem had stumped professional researchers for decades, galvanizing new algorithm improvements learned from human strategies (Khatib et al., 2011). More recently, as Google Deepmind's AlphaFold program improves its predictions of protein structure, the Fold.it community has shifted its focus from structure prediction to protein design and to fitting electron density (fold.it/portal/node/2010912).

Problems in music research, especially those having to do with ill-defined areas of music engagement and musical creativity in performance and in composition, are computationally difficult to solve; algorithmic solutions to these problems often yield partial and unsatisfactory results. Participation in citizen cyberscience, projects utilizing technology, come in the form of volunteer computing (sharing computing resources), volunteer thinking (assisting in cognitive tasks according to prescribed protocol), and participatory sensing (distributed data collection) (Haklay, 2013). A key component of these successful projects is their **ability to effectively harness citizen science, in particular, volunteer thinking for scientific research**.

Most citizen science projects have been based primarily on visual recognition or on manipulation of graphics; audio centric projects are still rare. For example, on Zooniverse (zooniverse.org), the only two audio projects have been based on identification of animal sounds in WhaleFM (Shamir et al., 2014) (github.com/zooniverse/WhaleFM) and Bat Detective (Aodha et al., 2018). Music science experiments frequently require empirical data from listeners and large empirical studies exist. The SALAMI (Structural Analysis of Large Amounts of Music Information) project (DDMAL, 2019) employed 8 music graduate students to provide the 2,400 structural annotations of approximately 1,400 recordings (Smith et al., 2011). Leech-Wilkinson and Prior (2014)'s study of shape in classical music performance recorded feedback from 189 musicians; Farbood (2012)'s web-based empirical study on perceived tension shapes enlisted 2661 participants from 108 countries to annotate short music snippets; and, Kaneshiro et al. (2017)'s study analyzed 188,271,243 Shazam queries across top 20 billboard popular songs. The opportunity exists to study music performance across structural and perceptual perspectives on a large (web) scale using similar principles of citizen science.

Factors such as recognition, game play elements, and team play have been found to help sustain engagement in citizen science projects (Jennett et al., 2016). In the context of music performance, this could mean focusing on highly engaging aspects of music experience and on elemental tasks such as noting change, recognizing and making tipping points (musical thresholds), and identifying or deciding on figural groupings. In his seminal book on emotion and meaning in music, Meyer (1956) argued that emotion response to music arises from the composer's choreography of expectation. Musical expectations–the anticipations they engender and the pleasure that arises from attaining the abstract musical reward–have been linked to dopamine release (Salimpoor et al., 2011). Further research has shown that music listening uses the same reward pathways as food, drugs and sex to induce pleasure (Mallik et al., 2017). It thus stands to reason to use this biological advantage of music, something that performers and composers have exploited for eons, in the design of engaging musical tasks for music citizen science projects.

### 2.4. Exploratory Tools: Gamifying the Thinking Behind Performance

Participant engagement in citizen science projects has been linked to opportunities for learning and creativity (Jennett et al., 2016), and gamification is increasingingly used to motivate and sustain engagement (Eveleigh et al., 2013). Can we create online sandbox environments for experimenting with making authentic performed music structures based on an understanding of music structures? A goal would be to provide engaging game play environments with authentic problems (based on what practitioners do) for exploring performed structures, giving participants different ways of representing the problem. A suite of online music performance exploration tools could allow participants to make performed structures such as tipping points and figural groupings, deciding where to place them and the articulations or other expressive deviations to use. When people participate in creating performed structures, they are then constructing and articulating their understanding of how that structure works.

Influential learning theories include Papert and Harel (1991)'s constructionism, where learners make mental models to understand their environment, and Piaget (1964) constructivism, where people actively construct their knowledge based on their own experiences. Examples of of constructivist-constructionist design in music games include, Bamberger (1974)'s Music Logo, a tool for building elementary algorithms to construct structures such as musical sequences, and its successor, Impromptu (Bamberger, 2000), an exploratory composition tool based on assembling and manipulating tune blocks (melodic fragments) to foster a project-based approach to making and understanding music (Bamberger, 2015). Another example, Earsketch (earsketch.gatech.edu), is a software tool for learning coding by remixing music audio snippets (Freeman et al., 2014). Earsketch has been used by 60,000 students in over 100 countries.

Widespread access to unprecedented computing power means that music (video) games with real-time control are now part of the digital landscape. While many music performance games exist in the form of Guitar Hero (Egozy, 2016) and Magic Piano (www.smule.com), etc., these designs largely judge a user's game play by how well they fit a template performance. As such, they function effectively as target practice modules: the next note plays when a button is pressed or screen tapped at the right time. The latest in Harmonix's suite of games, Fantasia Evolved, adds an “expressive” element only in that it allows users to improvise melodies and rhythms from a limited control palette.

Music games that allow expressive control over music parameters like tempo and loudness are broadly categorized as conductor programmes, whether or not they involve conducting gestures (Malinowski, 2007). Many use actual conducting gestures, or archetypal forms of the gestures, to control audio playback; a number of such systems exist as museum installations. In the Personal Orchestra (Borchers et al., 2004) at Vienna's Haus der Musik, conducting motions control the video and audio playback of a performance by the Vienna Philharmonic. The Mendelssohn Effektorium at Leipzig's Mendelssohn-Bartholdy Museum lets the user conduct, using a baton, an orchestra of free standing speakers each representing an orchestra section; the setup affords the conductor loudness control over individual orchestra sections. Numerous handheld (including mobile) music conducting games exist like the Nintendo Wii games, which includes a music module that allows users to conduct an orchestra of Mii avatars by waving the Wii remote controller like a conductor's baton (Bott et al., 2009).

Max Mathews (1991) created an early conducting interface, the Radio Baton, that lets the user control the timing of beats and loudness parameters. In Rasamimanana et al. (2011), players advance the music one beat every time a chess piece is moved or a ball tossed and caught. In the Air Worm (Dixon et al., 2005), a recording is stripped of its tempo and loudness variations, and users can re-insert expressive variations by moving a hand over a theremin configured to represent Langner's two-dimensional tempo-loudness space (Langner and Goebl, 2002). The Director Musices (DM) system gives users direct control over 30 rules covering different aspects of music performance, such as note duration and amplitude (Sundberg et al., 1983; Friberg et al., 2000); rules can be combined to create different desired affects. The successor pDM, allows real-time control of the DM rules (Friberg, 2006). In Music Plus One and the Informatics Philharmonic (Raphael, 2010, 2014), a machine tracks the soloist's position in the score and warps a recording of the orchestra part to match her/his paces; here, the soloist acts as the conductor. In an AI approach to expression synthesis, Widmer (1995, 2002) proposes to synthesize expressive performances according to the rules learned from data; this is further developed in Flossmann et al. (2009) and Flossmann and Widmer (2011).

Performing music operates on metaphors because sound itself has a small descriptive vocabulary. Leech-Wilkinson and Prior (2014) gives the example: we do not say, “increasing power in the upper quartile of the frequency spectrum is matched to decreasing inter-onset intervals and increasing sound pressure as the fundamentals of the singer's note sequence increase in [cycles per second],” rather we say, “the color brightens as the line surges upwards.” We speak of trajectories and landscapes when we describe music performance. Listeners of tonal music have been found to understand, experience, and create music (in part) through a **metaphorical process that maps physical to musical motion** (Todd, 1992), and that motion is shaped by analogies to gravity, magnetism, and inertia (Larson and Vanhandel, 2005). Chew et al. (2005) proposed that performing a piece of music is very much like driving a car, especially if one is a pianist, in the seat of a powerful machine, depressing the pedals, and modulating the speed of travel and the dynamics of the motion. Admittedly, there are limits to this analogy. Taking this metaphor literally and combining elements of game play and music structure scaffolding, ESP (Expression Synthesis Project) provides a driving (gas/brake pedals and wheel) interface for non-experts to navigate through a road representing a path through a musical piece (Chew et al., 2005; Liu et al., 2006), see (vimeo.com/231258088).

### 2.5. Computational Music Cognition: Duality Theory

The explosion and global impact of digital music information has led to a critical need for new computational tools and methods for **music information retrieval** (MIR) tasks such as music processing, analysis, organizing, search and access, and interacting with music data. The International Society for Music Information Retrieval (www.ismir.net) was founded in 2,000. There is unprecedented demand for nuanced but efficient and scalable computational tools for exploring, understanding, and discovering music structure.

Having an effective and accurate **representation of music structure** allows vast amounts of digital music to be indexed (Pienimaki, 2002; Chai and Vercoe, 2003), summarized (Logan and Chu, 2000; Cooper and Foote, 2003; Grosche et al., 2012), and retrieved (Martin et al., 2009) more efficiently. Formal representation also enables automated reasoning about music structures, in tasks such as chord transcription (Mauch et al., 2009), similarity assessment (Mardirossian and Chew, 2006), and music categorization (Tzanetakis and Cook, 2002). Representations that closely align with human perception of music structures lead to better retrieval results. More developments in computational music structure analysis are covered in Müller et al. (2016). Music structure in MIR most often refers to sectional form, with the task of structure analysis simplified to identifying boundaries and assigning labels indicating similar sections. Methodologies for annotating music corpora (Peeters and Deruty, 2009; Smith et al., 2011; Bimbot et al., 2012) and for evaluating structural analyses (Lukashevich, 2008; Nieto et al., 2014; McFee et al., 2015) have become important subtopics in MIR. Music corpora annotated with structure information still privilege abstract compositional form and not actual experienced or performed music structures.

**Computational music structure analysis**, the design of computer-based techniques to automatically determine music structure, forms one of the most crucial problems in MIR (Müller et al., 2016), with **predicting the structures listeners perceive** being one of the most popular tasks (Foote, 2000; Peeters, 2004, 2007; Paulus and Klapuri, 2006; Shiu et al., 2006; Kaiser and Sikora, 2010; Paulus et al., 2010; Hargreaves et al., 2012). The predominant approach takes music data or features and imputes a singular structure to that input. But, human perception and experience of even rudimentary sectional structure can vary widely for the same music material, depending on factors such as prior knowledge, attention, expectation, level of information, and ontological commitment (Smith et al., 2014). Thus, solving directly for structure given music data is not only difficult, the solution itself can be a moving target.

Mathematical optimization problems can be viewed from one of two related perspectives, the primal or the dual (Hillier and Lieberman, 2001); often, one is significantly easier to solve than the other. Drawing upon this idea, in Smith and Chew (2013, 2017), we turned the music structure analysis problem on its head to focus on **explaining why a listener perceives a particular structure**. In this work, we made perceived structure the given data, and solved for dynamically varying attention to different features to explain the perceived structure, for example, what features could the listener have been paying attention to so as to have perceived a boundary (a change) at a moment in time. This inverse problem is much easier to solve, see Smith and Chew (2013, 2017), and provides perceptually meaningful insight into any given interpretation of music structure. Another advantage and novelty of invoking the duality principle is that it changes the variables, and allows multiple analyses to be modeled each as a function of the corresponding listener, rather than having one prototypical analysis be the function of the music data.

*Invoking duality or solving the inverse problem represents a paradigm shift*. The **duality principle** generalizes to any music structure, and any concurrent information such as cardiac response, and opens up new possibilities for computational music structure analysis and computational music cognition.

### 2.6. Music Perception and Cognition: Computational Thinking

As a complement to MIR's focus on sectional structure, music psychology, cognitive science, and music theory have concentrated on the cognition of basic musical structures such as pitch entities—pitches, chords and keys (Krumhansl, 1990; Lerdahl, 2001) or melodies (Narmour, 1990, 1992)—and time structures—rhythm and meter (Lerdahl and Jackendoff, 1983; Hasty, 1997; Temperley, 2001; Mazzola, 2002) cover all three. Many of these theories are amenable to computer implementation, but much more is needed to **optimize the methods and representations to ensure accuracy, robustness, efficiency, and scalability**.

These aspects of modeling music perception and cognition have to do with **computational thinking**, a term coined by Seymour Papert (1980) and popularized by Jeannette Wing (2006) to describe the adoption of ways of formulating problems and expressing their solutions so that a computer (human or machine) can effectively carry it out. The following three examples illustrate the impact of computational thinking on models of music perception and cognition.

*1) Using contig mapping to boost voice separation performance:* Bregman (1990)'s theories on auditory scene analysis (Gestalt principles for sound) inspired Huron (2001) to derive voice-leading rules from perceptual principles. (Chew and Wu, 2004) operationalized Huron's voice-leading rules in the Voice Separation Analyser (VoSA) using a contig mapping approach.

VoSA's algorithm separates the voices from a polyphonic texture by taking advantage of voice-leading perceptual principles and by applying a contig mapping approach. In computational biology, contig mapping is a technique used to re-construct a DNA sequence from its fragments. The basic ideas behind the VoSA algorithm are as follows: because voices tend not to cross, when all voices are present, the order of the voices is known; these regions where all voices are present form maximal voice contigs and serve as islands of certainty where the voice assignments are known. Since voices tend to move by step, the voice assignments then grow out of the maximal voice contigs like tentacles by connecting to nearby notes. The algorithm is efficient, growing the islands progressively with each left-to-right then right-to-left scan, until all notes are labeled and islands joined. This algorithm was evaluated on Bach's *Two-* and *Three-part Inventions* and on fugues from the *Well-tempered Clavier* and Shostakovich's fugues from the *24 Preludes and Fugues*. Other researchers (Ishikagi et al., 2011; Guiomard-Kagan et al., 2016) have built on this idea of contig mapping.

The VoSA contig-mapping algorithm has remained one of the best performing voice separation algorithms to this day, serving as the benchmark for other voice separation algorithms, as shown in an independent comparative study (Guiomard-Kagan et al., 2015), which evaluated several algorithms on Bach's fugues from the *Well-tempered Clavier* and a corpus of popular music containing polyphonic information.

*2) An interior point approach to modeling tonality:* Mental representations of pitch structures forms one of the oldest topics in music perception (Lerdahl, 2001). Following early studies by Shepard (1982) on mental representations of musical pitch, Krumhansl (1990) proposed several empirically derived models of pitch structures using multi-dimensional scaling (Shepard, 1962a,b). Parallel developments in cognitive science saw the advent of the harmonic network, which Longuet-Higgins (1962a,b) based on frequency ratios of musical intervals; the resulting spatial representation is isomorphic to the neo-Riemannian tonnetz (Cohn, 1997). The tonnetz has been used to track harmonic motion in romantic (Cohn, 1996), classical (Bragg et al., 2011), jazz (Briginshaw, 2012), and popular/rock (Capuzzo, 2004; Chuan and Chew, 2011) music. The harmonic network was used in an early key finding algorithms (Longuet-Higgins, 1971). A widely used result in (Krumhansl, 1990) is the key profile correlation approach to identifying key, and was improved in Temperley (1999, 2001, 2006) and applied to music audio (Gomez, 2006).

Interior point methods are a class of algorithms that search for optimal solutions to linear and nonlinear convex optimisation problems from within the feasible region. Inspired by research with Dantzig (1992) on von Neumann's early interior point method which traversed the interior space rather than pivoting through a sequence of corner point solutions, I proposed an interior point approach to modeling tonality and key finding (Chew, 2000, 2014). The spiral array model drew upon the insight that the harmonic network is essentially a three-dimensional helical model, and used the space inside the helical pitch class model to summarize the Center of Effect (CE) of pitch collections and to successively build the nested helices that make up the spiral array model. The model differs from its predecessors in that it represents pitches, chords, and keys in the same space; this allows for the comparison of tonal entities within the same and across different hierarchical levels. The spiral array's Center of Effect Generator (CEG) key-finding algorithm has been shown to outperform prior techniques and, in practice, tends to yield solutions within only a few iterations (Chew, 2001). The algorithm can also be adapted to the tonal analysis of music audio (Chuan and Chew, 2005). This model is regarded as one of the most successful models for tonal perception.

*3) Modeling tension modulation over time and applications to music generation:* Sensations of music expectation, tension, surprise (delightful or otherwise) are key sensory aspects of musical experiences. Huron (2004) showed, in a systematic analysis of the humor devices in the music of PDQ Bach, that strong emotion responses like laughter result from violations of expectations. Inspired by this research, Chew (2001) visualized the violations of tonal expectations using MuSA.RT (Music on the Spiral Array, Real-Time), an implementation of the spiral array model with real-time visualization of its tonal analysis algorithms–see bit.ly/ec-MuSART and bit.ly/ec-LAPhil. Tonally consistent music passages map to closely clustered CE trails; tonally unexpected events lead to distant detours.

Tension has been found to result from states of conflict, instability, uncertainty, and dissonance (Lehne and Koelsch, 2015). Quantitative models of musical tension include (Lerdahl and Krumhansl, 2007; Farbood, 2012; Herremans and Chew, 2016). (Lerdahl and Krumhansl, 2007) is based on Lerdahl (2001)'s theory of tonal tension. Farbood developed a model based on findings from a large-scale empirical study of listener annotations of perceived tension for small musical snippets. Our model uses three quantitative measures based on the spiral array model—the cloud diameter (dissonance), cloud momentum (an indicator of uncertainty), and tensile strain (a mark of instability); these tension quantifiers were tested on piano sonatas by Beethoven and Schubert (Herremans and Chew, 2016).

Tension models are important tools for guiding computer music generation. With the growing popularity of deep learning and the rise of the Google Magenta project (magenta.tensorflow.org), interest in generating music and art using machine-learning techniques has reached new heights. A review of music generation systems can be found in Herremans et al. (2017). However, machine-generated music is notoriously devoid of long-term structure or narrative interest. Some attempts have been made to address this problem through design of structural control into improvisation systems (Dubnov and Assayag, 2005; Francois et al., 2013; Schankler et al., 2014; Assayag, 2016; Nika et al., 2016). In Hyperscore (Farbood, 2001) a user-defined tension line controls the dissonance of the generated music. In MorpheuS (Herremans and Chew, 2019), the time modulating tension profile learned from a template piece constrains the optimisation-based music generation algorithm to impose some narrative structure. The output is also constrained to copy the rhythms of the template piece; because repetition is a key feature of music (Margulis, 2013), the output must also adhere to the template piece's structure of repeated patterns. The resulting piece thus inherits multiple types of structure from the parent piece to produce usable and interesting polyphonic output from one single template piece–see dorienherremans.com/morpheus/.

A theme running through these examples is the *use of computational thinking to transform solution approaches to music structure modeling and analysis in ways that improve accuracy, efficiency, and scalability*.

## 3. Musical Structures in Electrocardiographic Signals

Computational arrhythmia research has flourished due to the explosion in cardiovascular information and widespread adoption of equipment for collecting and analyzing ECG data. Globally, ECG equipment and management systems were valued at 5.6 billion USD in 2019, with a compound annual growth rate of 6.1% from 2020 to 2027 (Grand View Research, 2020). Consumer level ECG recording and analysis devices are becoming increasingly popular and accessible. Wearable, mobile devices like the Apple Smartwatch, now come equipped with electrical heart rate sensors that can record wearers' electrocardiograms (ECG) and can detect if the heartbeats are normal or in atrial fibrillation—fast and irregular rhythms originating in the upper chambers of the heart. Here, we advocate for transferring the computational models and experiential approach developed for the analysis of performed music to heart signals. We make the case for analyzing arrhythmia recordings as one would musical performances–attending closely to the details and nuances of time structures and time varying structures, to their evolution over time, to the nature of transitions into and out of states of arrhythmia–in a human-centered approach.

### 3.1. Similarities Between Music and Heart Signals

Computational music analysis techniques translate well to the analyzing of heart signals because music and heart signals share many common behaviors and structures. These commonalities in time and frequency structures between music and the heart mean that the language and methods for describing musical structures can be applied to many aspects of cardiac signals.

The links between the musical pulse and normal cardiac rhythms have been observed historically (Siraisi, 1975). Like in music, the heart never beats in uniformly equal time intervals; heart rate variability is a sign of a healthy heart. An interesting observation is that a heart on a pacemaker produces beats like music on a click track. Beat-to-beat rhythmic variations have been found to exhibit 1/*f* (fractal) behavior in not only composed music (Levitin et al., 2012) but also performed music (Rankin et al., 2009; Räsänen et al., 2015). Similar long-range (1/f type) correlations have also been found in cardiac signals (Goldberger, 1992), which show the heart's performed heartbeats when beating normally. Similarities between music and arrhythmias also occur at larger time scales. Arrhythmias are often episodic, with music-like sectional form. Most episodes begin with a trigger like an early ventricular beat that acts as an upbeat into the arrhythmic section that is performed differently every time.

It is the pathophysiology of cardiac arrhythmia that gives rise to the most interesting and striking parallels between music and heart rhythms. Suppose a heart in sinus (normal) rhythm is a performer playing a rhythm close to a score of equi duration, say crotchet, notes. Then the heart in a state of arrhythmia is playing a wayward performance of this score. A computational approach that places emphasis on the performed musical structures can thus be applied to the analysis of its eletrocardiographic (ECG) signals. In the time domain, consecutive inter-beat intervals in ECG traces of arrhythmias exhibit a propensity to form simple ratio categories (Chew et al., 2021), leading to observed parallels between the rhythms of arrhythmia and notated music (Goldberger et al., 2014; Chew, 2021). These duration ratios do not fall on precise proportions like 1:1, 1:2, etc. Like in performed music, they may come close, but avoid the exact ratios themselves.

In the frequency domain, the dominant frequency which varies over time is an important characteristic of atrial fibrillation sequences, like the time varying fundamental frequency in performed music. Fibrillatory waves between heartbeats in atrial fibrillation are like musical vibratos. The amplitude and stability of these vibrato-like fibrillatory waves are markers of disease progression (Meo et al., 2015; Mishra et al., 2019). Thus, analyzing these fibrillatory waves like one would musical vibratos like in Yang et al. (2017), can reveal detailed behaviors to inform disease stratification and treatment.

### 3.2. Notating Heart Rhythms

Notational technology evolved over the centuries to allow performers to replicate music note sequences faithfully (Kelly, 2014). Traditional music notation was used to accurately transcribe the extreme rhythms of music performance in Chew (2018). The difference between what the composer wrote (the abstract idea) and what the performer did (the actual experience for the listener) was made all the more stark by the use of the same notational technology. Taking advantage of this flexibility of music notation to (unconventionally) represent performed variations, music notation has also been used to represent cardiovascular pathology.

The first use of music symbols to represent a heart murmur dates back to René Laennec (1826), inventor of the stethoscope, who used it to track a venous hum heard in auscultation (Segall, 1962). More recently, Field (2010) used music notation to transcribe signature heart sounds and murmur patterns in the teaching of cardiac auscultation.

**Figure 2:**
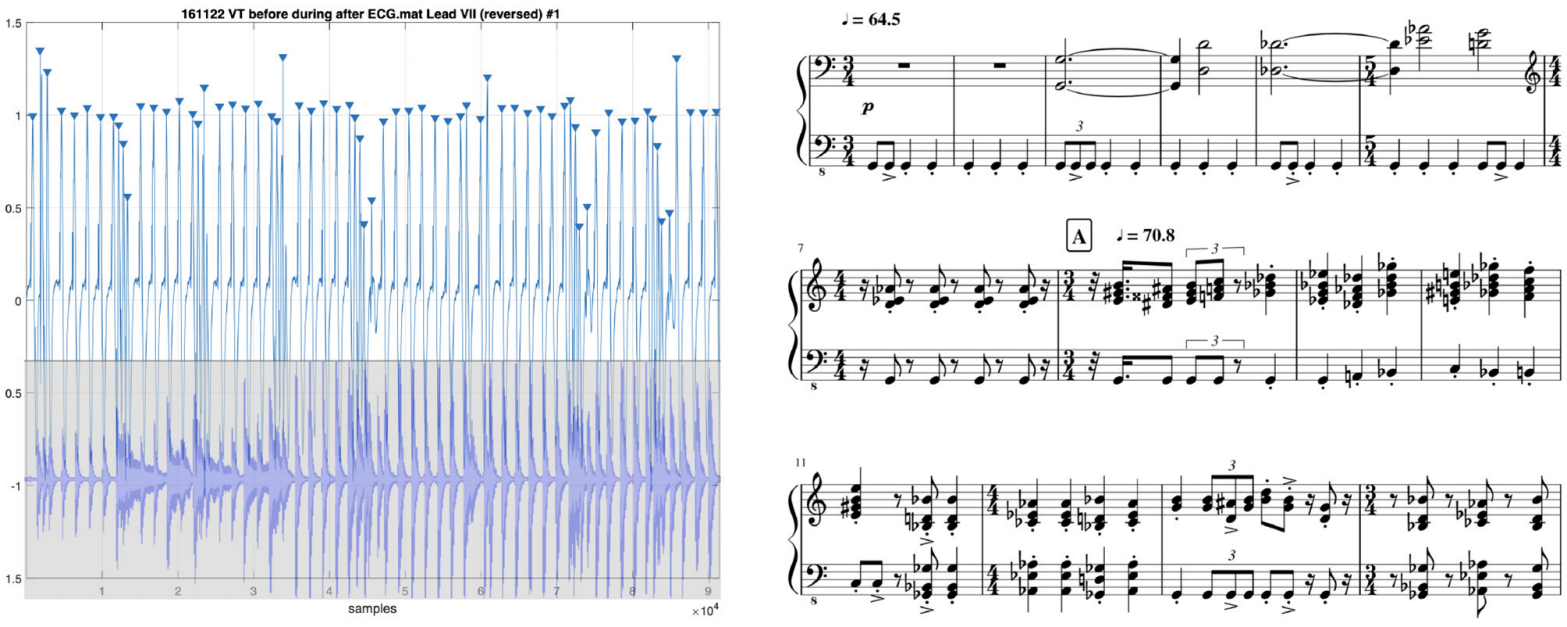
Arrhythmia suite I: VT before during after (2017), after Mars, the bringer of war (1914), from the Planets, Op.32. Composition by Holst/Chew, Krishna, Soberanes, Ybarra, Orini, and Lambiase. **(Left)** ECG signal overlaid with audio of performance of **(right)** beginning of piece.

Music symbols can be used to represent not only the sounds heard through a stethoscope, but also electrical anomalies recorded in ECGs whose abnormal rhythms may be felt in the pulse. Noting similarities between music and aberrant heart rhythms, Goldberger et al. (2014) hypothesized that the dotted rhythm at the beginning of Beethoven's “Adieux” Sonata (Op.81a) might be ascribed to the composer's possible arrhythmia. Chew et al. (2021) added to this a possible arrhythmia-based inspiration for the opening motif of Beethoven's Fifth Symphony. Chew (2018) used music notation to represent the rhythms of atrial fibrillation, employing mixed meters and metric modulations to accurately capture the high rhythmic variations. This method has been used to compose pieces in the *Arrhythmia Suite* (Chew, 2021)—see excerpt and corresponding ECG in Figure 2. A performance can be heard at youtu.be/z8aspgwes1o. Such **direct mappings between heart and music rhythms suggest an avenue exists for the transfer of computational music structure analysis techniques to cardiac signals**.

Similar to this use of symbolic notation to capture the variations of an ECG sequence, Syed et al. (2010) and Qiu et al. (2016) studied the semantic structure of symbols representing ECG waveform parts. Bettermann et al. (1999) used a binary symbol sequence to represent elementary rhythm patterns in heart period tachograms.

Further capitalizing on the link between music and heart rhythms, musicians and scientists have used heartbeat-to-music mappings to generate new music and for medical diagnosis. Goldberger et al. (1995) mapped beat intervals averaged over 300 beats to a diatonic scale to create melodies for musical improvizations. Other researchers have mapped beat intervals to MIDI notes with premature beats producing more significant pitch changes (Goldberger et al., 2002), or to MIDI note onsets, pitch, or loudness (Orzessek and Falkner, 2006). Ballora et al. (2006) mapped heart rate variability indices to pitch, timbre, and pulses over hours for diagnosis. The Heart Chamber Orchestra (Votava and Berger, 2011) used interpretations of its 12 musicians' heartbeats to generate a real-time score which the musicians then read from a computer screen and perform.

### 3.3. Computational Arrhythmia Research: Musical Thinking

The examples shown in the previous sections support the case for a musical approach to ECG analysis, particularly one that emphasizes individual and specific experiences of an arrhythmia. Rather than focusing on coarse categories, this experiential approach treats each ECG recording like a unique performance. This is possible because the vicissitudes of cardiac arrhythmia, and even that of normal heartbeats, are similar to that introduced in performance. Thus, techniques designed for characterizing performance variations and the musical structures therein translate well to cardiac signals, with potential for revealing patterns not detected by current methods.

Popular standard measures like the heart rate variability (HRV) (Billman, 2011; Ernst, 2014) are summary statistics or spectral indices that do not capture moment-to-moment details of temporal structures and the nature of the transitions between them. For example, the transition from normal to tachycardia states can be as varied and interesting as pianists' different ways of bridging the Grave and Doppio Movimento sections in Chopin's Sonate Op. 35 in B minor. Furthermore, many arrhythmias can often be episodic in nature, but the emergence of such long-term structures as the arrhythmia unfolds has not been a focus of analytical research in cardiology. On the other hand, the characterization of the shaping of these (and other) musical structures in recorded music performances is an essential aspect of the performance research– see for example (Stowell and Chew, 2013; Chew, 2016).

The implications of applying musical thinking to computational arrhythmia research go beyond the translation of computational music structure analysis techniques in cardiovascular science; it also presents a streamlined and unified analytical approach to studies on cardiac and music interactions in neurocardiology. For example, in Chew et al. (2020), the same analytical techniques were applied to both cardiac and music features to assess their coincidence in a study with pacemaker patients listening to live music performance. The complete range of music-heart applications is beyond the scope of this article.

## 4. COSMOS Research Objectives

To achieve the trans-disciplinary goals outlined above, the COSMOS project sets out three mutually-informing research directions. The first is to research problem solving skills in the perception and cognition of musical structures, taking advantage of crowdsourcing to amass data requiring human cognitive abilities. The second aims to investigate decision making in the act of shaping performed structures through software-based sandbox environments. The third objective is to develop a novel methodological framework for computational music structure analysis in the context of music performance, with extensions to cardiac signals.

The research themes are given below, each described in greater detail in the subsections to follow:

- to find new ways to represent, explore, and talk about the musical structures in performance and performed music, and other music-like sequences such as cardiac signals;
- to harness human problem solving skills through citizen science to understand musical structures created in performance and experienced in performed music;
- to create sandbox environments to experiment with making performed structures;
- to create theoretical frameworks to discover the reasoning behind or explain the structures perceived, sensed, and made;
- to foster community engagement by training experts to provide feedback on structure solutions.

Analysis of the perceived and designed structures will be based on the inverse problem paradigm of duality to reverse engineer and explain why a listener or analyst perceives, or a performer chooses, a particular structure. Embedded in the approach is the use of computational thinking to optimize representations and theories to ensure accuracy, robustness, efficiency, and scalability.

The project, which aims to reconfigure the way researchers and the general public view music performance, will draw from a broad range of recorded piano performances from different times having accurate timing, dynamics, and articulation information. The methods will also be applied to cardiac recordings, particularly those of arrhythmia, to provide descriptors to aid in cardiac diagnostics and therapeutics.

### 4.1. Musical Structures From Performance and Cardiac Signals

Performances and music-like sequences such as cardiac signals form an untapped source for music structure analysis (Chew, 2017, 2018) in music information research and research in music perception and cognition. This theme focuses on extracting and representing the experienced music structures in recorded performances and arrhythmic cardiac signals. The goal is to create new ways to represent and talk about the variations and structures introduced during performance, to encourage multiple representations and abilities to move among them. For music, this will offer new ways to encode, explore, and reason about performance decisions.

For music, the research questions include: What are the felt and experienced structures in performed music? How are these structures generated? How do they differ from the formal structures embedded in the score? What music features do they serve to highlight? Are there parts of a piece more open to multiple structural interpretations and parts that have limited possibilities and why? For cardiac signals, the corresponding research questions include: What are the felt and experienced musical structures in cardiac arrhythmias? What is the range of individual experiences of different arrhythmias? Does the musical representation reveal patterns and trends that were not previously apparent? Can the variations be linked to symptoms or outcomes?

### 4.2. Harnessing Volunteer Thinking

Reporting on sensory aspects of music experience, such as feelings of tension, has been found to be amenable to web-based empirical research (Farbood, 2012). This theme adopts best practices in citizen science (Vohland et al., 2021) to engage non-professionals (as well as seasoned experts) to participate in large-scale music performance research. Recognition and game play elements will assume important roles in the design of the cognition and problem solving activities. The focus will be on authentic problems, the kinds of problems that music practitioners face. Tutorials with worked examples by professionals will help enhance flexible thinking and reasoning about solutions. Scalable computational tools will provide multiple representation schemes–for example, tempo and loudness information overlaid on audio signal and spectral information—for cognitive scaffolding (Jonassen, 1999). Development will leverage existing online citizen science platforms and build on successful citizen science project designs. Questions include: to what extent can volunteer thinking benefit music performance research? What are the felt music structures best suited to this mode of investigation? What kinds of training and cognitive scaffolding should be provided so that non-professionals can participate productively? How can we ensure reliability of the data collected? How can we ensure broad participation and sustain engagement?

### 4.3. Sandbox Environments: Making Music Structures

Another series of activities will engage volunteers in the making of music structures in game play environments. The space of expressive possibilities of each piece of music is combinatorially large, and the unexpectedness, inevitability, and economy of elegantly reasoned solutions found in this space still confound machines. AI's may have beaten humans at games of chess and go, but there are still many problems that are easy for humans but computationally extremely difficult for machines; the design of CAPTCHAs (von Ahn et al., 2003) are based on this very principle. Participants of citizen science projects have made important discoveries–spotting new galaxies (Banfield et al., 2016)) in astronomy and protein folding (Cooper et al., 2010) in biology. We will design sandbox game play environments that call on volunteer thinking to explore new performance paths, or strategies for devising new performance paths, through music pieces. The sandbox environments will use a combination of symbolic and audio representations and audio features, and musical metaphors.

### 4.4. Perception Analytics and Design Analytics

This theme seeks to develop theoretical frameworks to analyse the data from structure perception, structure sensing (e.g., cardiac response to musical structures), and structure making tasks. The problem is to explain a set of annotations, responses, or design choices based on the underlying musical information.

The objective is to design new data-supported ways to explore, understand, and reason about musical structures, listener responses, and performance choices. A goal will be to increase understanding of the creative problem solving enacted in performance. For example, given a time modulating perception of tension or measure of physiological stress, we can reverse engineer what parts (fractions) of that perception can be attributed to dissonance, uncertainty, or instability using models such as Herremans and Chew (2016); given a (perception of) tonal structure projected in performance, we can reverse engineer the time-varying amount of prior information needed to reach that assessment using models like (Chew, 2002); given (a perception of) a series of boundaries, we can reverse engineer the changing attention to different audio features that gives rise to that perception (Smith and Chew, 2017).

Research questions this theme will address include: How can we model time-varying perceptual or sensory attributes like attention? What can these solutions tell us about the musical structures that produce the perceptual or sensory information stream? What does this tell us about the performer's or the designer's choices, or the perceiver's reactions?

The computational modeling will place emphasis on explanatory models, models for which parameters are transparent, and where it is possible to reason and step through the solutions. Another aim will be to choose models that are computationally scalable and efficient so as to be able to deploy them at scale. Adopting the principle of Occam's razor, simplicity should not be a deterrent, and will determine choices between competing hypotheses or theoretical models.

### 4.5. Community Engagement

This theme is modeled after the Worldwide Telescope Project (Goodman et al., 2012), which provides a platform for school students to access current astronomy research data (images of space) to design tours through the galaxies. The project trains astrophysically-literate volunteers (including retired astronomers) to provide feedback on the student-designed tours and answer questions about galaxies. The outcomes of the current project will feed into a worldwide web platform for exploring the perceiving and creating of music structures in performance. Retired musicians, music teachers, conservatoire students, and other musically literate volunteers will be trained to provide feedback on structures perceived and made by participants, so as to increase public understanding of the creative work in music performance.

## 5. Conclusion

In conclusion, this position paper has presented the view of music performance as being centered on the search and rendering of musical coherence, and provided supporting evidence for the role musical structure plays in performed music and the musical experience. The goal here is to instigate new ways of thinking about music performance, and of framing music information research (MIR) problems pertaining to the inter-related areas of music performance, performed music, and musical structures.

The following principles were put forth to link music performance and music experience to musical structure: the role of musical structure in music engagement keeping in mind the role of music performance of creating engaging experiences; a view of creative performance as problem solving; the harnessing of volunteer thinking in citizen science to find structures; the gamifying of performance decisions to enable learning by doing; reverse engineering the reasons for interpretive decisions, structure perceptions, or other concurrent information such as cardiac responses; and, the case was made for the incorporation of computational thinking into music structure modeling and analysis, and for applying musical thinking to computational arrhythmia research.

Much of this approach applies also to other music-like sequences. Leveraging the structural similarities between heart and music signals and the inter-transferrability of analysis techniques, cardiac electrophysiology was suggested as an area for MIR exploration, with potential to provide insights into cardiac arrhythmia and analytical approaches for music-heart studies.

Based on these principles, a set of five research objectives are proposed: to find new ways to represent and talk about performance; to use citizen science to engage experts and everyday listeners in the problem solving task of apprehending musical structures in performance and performed music; to create sandbox environments to enable people to explore the space of possible interpretations; to design theoretical frameworks to uncover the reasoning behind musical structures perceived, sensed, designed, and made in performance; and, to increase public awareness of the creative work of performance by fostering community engagement to provide feedback on structures found and made.

The ERC project COSMOS aims to address the research issues raised here, focussing on performed music as well as cardiac recordings as applications of computational music structure modeling and analysis. A purpose of sharing the assertions and goals of the research is to galvanize and encourage others to join in these efforts. These problems transcend the project with potential to benefit artistic, scientific, and medical knowledge about music performance, processes of perception and design, variations in individual choices and experiences in music and arrhythmias.

## Author Contributions

EC conceived, designed, wrote, revised the article, and agrees to be accountable for all aspects in ensuring that questions related to its accuracy or integrity are properly investigated and resolved.

## Funding

EC was supported by the European Research Council (ERC) under the European Union's Horizon 2020 Research and Innovation programme (Grant Agreement No. 788960).

## Conflict of Interest

The author declares that the research was conducted in the absence of any commercial or financial relationships that could be construed as a potential conflict of interest.

## Publisher's Note

All claims expressed in this article are solely those of the authors and do not necessarily represent those of their affiliated organizations, or those of the publisher, the editors and the reviewers. Any product that may be evaluated in this article, or claim that may be made by its manufacturer, is not guaranteed or endorsed by the publisher.
